# Prevalence of high-grade perineal tear during labor in Mexican adolescents

**DOI:** 10.25100/cm.v49i4.3515

**Published:** 2018-12-30

**Authors:** María Teresa Sánchez-Ávila, Marisol Galván-Caudillo, Jaime Javier Cantú-Pompa, Natalia Vázquez-Romero, Jhanea Patricia Martínez-López, Víctor Manuel Matías-Barrios, Abryl Mariana Avitia-Herrera, Luis Alonso Morales-Garza, Claudia Eugenia Hernández-Escobar, Gonzalo Soto-Fuenzalida, María Teresa González-Garza

**Affiliations:** 1 Tecnologico de Monterrey, Escuela de Medicina y Ciencias de la Salud Departamento de Ciencias Clínicas. Monterrey, Nuevo León, México.

**Keywords:** Episiotomy, lacerations, pregnancy in adolescence, rectovaginal fistula, delivery, obstetric, parity, pregnancy, Episiotomia, laceraciones, embarazo en adolescentes, fistula cectovaginal, parto, obstetricia, paridad, embarazo

## Abstract

**Introduction:**

There is a high rate of deliveries in adolescents in Mexico. This age group is vulnerable to obstetric complications, including lacerations of the anal sphincter.

**Objective:**

To determine the prevalence of third and fourth degree perineal tears in adolescents during childbirth, and to evaluate risk factors in comparison with deliveries with lacerations of adult women.

**Methods:**

All obstetric care episodes were reviewed from a public tertiary hospital data in Monterrey, Mexico in 2014. Age, primiparity, delivery instrumentation, episiotomy, body mass index, product weight and tear´s degree were documented at the deliveries with tears of third and fourth degree.

**Results:**

The prevalence of third and fourth degree tears of 2.0% was found in the general population, being adolescents the most affected with 2.5%. The unadjusted odds ratio of high-grade tears in adolescent females at delivery, compared to adult females, was 1.36 (95% CI = 0.99-1.86, *p*= 0.05). No difference was found when comparing risk factors among high-grade tear deliveries in adolescents versus adults.

**Conclusions:**

A higher prevalence than previous reported for high grade tears during delivery was found. The data suggest adolescence as a risk factor for high-grade tears during delivery.

## Resumen

**Introducción:**

En México hay una elevada tasa de partos en adolescentes. Este grupo es vulnerable para complicaciones obstétricas, entre ellas laceración del esfínter anal.

**Objetivo:**

Conocer la prevalencia de desgarros perineales de tercer y cuarto grado en adolescentes durante el parto y evaluar factores de riesgo en comparación con partos con laceración de mujeres adultas.

**Métodos:**

Se revisaron todas las atenciones obstétricas en un hospital publico de tercer nivel en Monterrey, Nuevo León, México en el año 2014. Se documentó edad, primiparidad, instrumentación del parto, realización de episiotomía, índice de masa corporal, peso del producto y grado del desgarro en los partos que presentaron desgarros de tercer y cuarto grado

**Resultados:**

Se encontró una prevalencia general de 2.0% de desgarros de tercer y cuarto grado y en adolescentes de 2.5%. La razón de momios sin ajustar de desgarros de alto grado en mujeres adolescentes en comparación con mujeres adultas fue de 1.36 (IC 95%= 0.99-1.86, *p*= 0.05). No se encontró diferencia al comparar factores de riesgo entre los partos con desgarro de alto grado en adolescentes contra adultas.

**Conclusiones:**

Se encontró una prevalencia mayor a lo reportado de desgarros de alto grado durante el parto. Los datos sugieren a la adolescencia como factor de riesgo para desgarros de alto grado.

## Introduction

It is estimated that the birth rate in adolescents (range from 15 to 19 years) is 49 per 1,000 inhabitants, which corresponds to 11% of the worldwide births ^1^, of which 90% occur in low- and middle-income countries ^2^. Mexico is the country with the highest prevalence rate of teenage pregnancies among the Organization for Economic Cooperation and Development (OECD) members ^3^ and is considered one of the countries with the highest rate of pregnancies in this age group ^1^. In 2015, there were 405,876 pregnancies in adolescents aged 15 to 18 in Mexico, representing 18.2% of all births in the country ^4^. The importance of adolescent pregnancy lies in the fact that it represents a risk factor for complications for the mother-product binomial ^2^. The risk for the newborn is premature age (<37 weeks), low birth weight, and APGAR score <7 ^5^ ^-^ ^7^. Furthermore, for adolescent women there is an increased risk of obstetric complications, among which are preeclampsia-eclampsia, postpartum hemorrhage, puerperal endometritis, systemic infections, increased use of episiotomy and perineal tears during delivery, the latter being the most frequent obstetric injury ^7^ ^-^ ^9^.

Primiparity is recognized as a risk factor for perineal tears ^10^, since up to 73% of primiparous women develop moderate perineal tears, and 1% to 19% of vaginal births occur with sphincter laceration, thus involving third or fourth degree tears. Another factor is a high BMI, as this increases the risk of macrosomia and instrumented delivery ^11^, that has a greater risk, whether forceps or vacuum extraction are used ^12^. Regarding the episiotomy, its performance during delivery increases the number of severe perineal tears, being mediolateral technique the least damaging ^13^. Specifically, in adolescent delivery, risk factors for perineal tears have been documented: primiparity, fetal position, gestational diabetes (which requires insulin for glucose control), and duration of the second stage of labor ^14^.

It is estimated that third- and fourth-degree tears occur in 0.8% of all vaginal deliveries in Mexico ^15^. Likewise, obstetric anal sphincter injuries are associated with short and long-term sequelae, mainly anal incontinence, rectovaginal fistulas, wound dehiscence, and abscess formation, which affect physically and psychologically patients ^11^ ^,^ ^16^ ^-^ ^18^. In addition, fistulas and fecal incontinence ^19^ ^,^ ^20^ are mostly due to inadequate reconstruction of the anal sphincter, which is associated with medico-legal problems and increased health care costs ^21^.

The objective of this study is to determine the prevalence of third- and fourth-degree tears in teenage deliveries and in the general population of a public tertiary level hospital in Monterrey, Mexico. In addition, to determine if it is a risk for the development of high-grade perineal tears. Furthermore, to assess whether adolescence is related to risk factors for delivery-related perineal tears compared to deliveries in adult women. who developed high-grade perineal tears,

## Materials and Methods

This is an observational and analytic study.

### Sample

We reviewed the records of women with vaginal obstetric care (January 1^st^ - December 31^st^, 2014) in the High Specialty Maternal-Child Hospital (Hospital Materno-Infantil at Monterrey, Mexico), which is a reference center that provides general and specialty obstetric care in the Northeast of Mexico. All cases were reviewed, but only cases with third- and fourth-degree tears were considered because of the anal sphincter involvement. Cases with presentations other than cephalic, multiple pregnancies, cesarean birth or premature delivery (<36 weeks of gestation) were excluded. From these cases, the following data was documented: delivery, episiotomy, weight of the product, mother's body mass index at the time of delivery and parity of the patient.

### Statistical analysis

Age cut-off value was set at 19 years old, considering adolescents those patients under 19 years old. The adult women (<19 old) population in the sample was used for comparison (SygmaPlot; Systat v. 12). Prevalence was calculated relative to the total number of vaginal deliveries for the study period. Data is presented as number and percentage or median and range where appropriate. To determine risk association, the unmatched odds ratio was calculated. The Fisher exact test and Mann-Whitney U test were used to compare qualitative variables and quantitative variables. Statistical significance was considered for *p* ≤0.05.

### Ethical aspects

This study was approved by the Investigation and Ethics Committee (IRB) of both the Hospital and the Medical School. All data was collected anonymously. The information gathered for the study was for exclusive research purposes and only the researchers involved had access.

## Results

**Figure 1:**
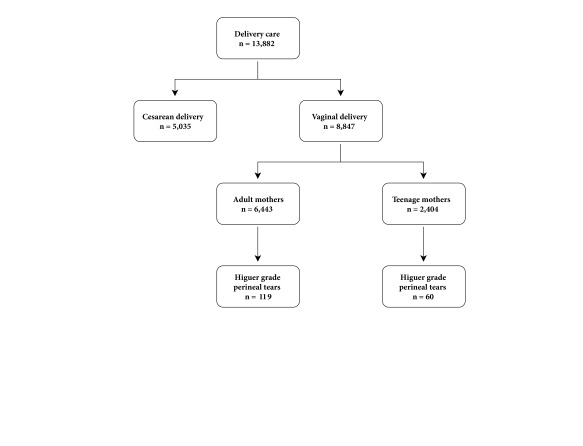
**Flow chart of case selection**.

During 2014, 13,882 births were cared for at the hospital during 2014. Of these, 8,847 were vaginal deliveries, 27% in adolescents (2,404 deliveries). A total of 179 cases of patients with third- and fourth-degree tears were identified, for a general prevalence of 2.0% during the study year. Adolescents accounted for 60 cases (33.5%), for a prevalence of 2.5% of tears in this age group (Fig. 1), higher than the prevalence of adult women (1.84%). The risk of high-grade tears was higher in adolescent deliveries in contrast to the births in adult women (OR= 1.36, 95% CI= 0.99-1.86, *p*= 0.05). 

Table 1 shows the results of the comparison of risk factors in deliveries with high-grade tear in adolescents versus adults. No significant difference was found when comparing the collected variables.

**Table 1:** Comparison of patients with high-grade tears with variables of interest

Variables	Patients groups		
	Adolescent	Adult	General population with high-grade tears
	(<19 years old)	(≥19 years old)	
Cases	60 (33.5%)	119 (66.5%)	179 (100%)
Age	17 (14-18)	22 (19-40)	20 (14-40)
Primiparity	56 (93.3%)	83 (69.7%)	139 (77.7%)
Instrumental delivery	48 (80.0%)	88 (74.0%)	136 (76.0%)
Episiotomy realization			
BMI	25.13 (17.80-39.43)	25.89 (18.90-48.39)	25.61 (17.80-48.39)
Product weigh (g)	3,390 (2,230-4,060)	3,270 (2,250-4,390)	3,300 (2,230-4,390)
3^rd^ degree tears	54 (90.0%)	108 (90.8%)	162(90.5%)
4^th^ degree tears	6 (10.0%)	11 (9.2%)	17 (9.5%)

## Discussion

In this study, a higher prevalence of high-grade perineal tears during delivery in the general population was found compared to that previously reported of 0.8% ^15^. This last work converged the experience of 21 centers in Mexico, representing a greater and more diverse sample. However, in five of these centers, third- and fourth-degree obstetric lacerations were not reported, which could imply a report bias (underreporting). As a possible explanation for the higher prevalence in our sample, is important to emphasize that the information is based on a single center. In addition, it may represent a selection bias since it is a reference unit for obstetric care of high complex cases for the population with public health services in the northeast of the country. We propose studies to allow for a better characterization of the differences between the prevalence and risk factors of obstetric perineal lacerations within the several regions of our country.

There are few studies where adolescent age is considered a risk factor for the development of high-grade tears. In the literature review carried out for the preparation of this manuscript, no studies were found that determined adolescent delivery as a risk factor for the Mexican population for this kind of pathology. Experiences in other populations supports adolescence as a risk factor for this complication ^22^, although some evidence suggest otherwise ^15^ ^,^ ^23^ ^,^ ^24^.

As a limitation of this work, it was not possible to perform an analysis where adolescence was determined as an independent risk factor for the development of high-grade tears during labor. In addition, although the result is statistically significant, the confidence interval of the crude odds ratio is very wide and is below unity. Even so, the data suggest that it is a group prone to this pathology and we encourage clinicians to be alert during the obstetric vaginal care of this age group to try to prevent this complication.

## Conclusions

In adolescent population, there is a higher prevalence of tears during vaginal delivery compared to adult women, and that reported in other studies in the Mexican population. The differences in the prevalence found compared to previous publications could be related to underreporting or selection bias. Likewise, the data suggest that adolescence could be a risk factor for high-grade tears. It is important to highlight the limited literature on perineal tears focused on the adolescent and Mexican population. The practical implications of these findings are to recognize adolescents as a vulnerable group for the development of high-grade perineal tears during delivery. Hence justifying the development of programs focused on decreasing the prevalence of high-grade perineal tears during vaginal obstetric care in our population, with special emphasis on adolescents.
