# Supplementary material for: Prevalence of high-grade perineal tear during labor in Mexican adolescents
Source: Colomb Med (Cali). 2018 Dec 30;49(4):261–4. doi: 10.25100/cm.v49i4.3515 (PMC6342085; doi:10.25100/cm.v49i4.3515)
Supplement: Related texts

# Original article: Prevalencia de desgarro de alto grado en parto de adolescentes mexicanas

## Introducción

Se estima que la tasa de natalidad en adolescentes de 15 a 19 años es de 49 por cada 1,000 habitantes, la cual corresponde al 11% de los nacimientos mundiales ^1^, de los cuales, el 90% ocurre en países de bajos y medianos ingresos ^2^. México es el país con la mayor tasa de prevalencia de embarazos en adolescentes en la Organización para la Cooperación y el Desarrollo Económico **(**OCDE) ^3^ y es considerado como uno de los países con una tasa elevada de este tipo de embarazos ^1^. En 2015, se registraron en México 405,876 embarazos en adolescentes de 15-18 años, representando el 18.2% del total de los nacimientos del país ^4^. La importancia del parto adolescente radica en que representa un factor de riesgo para complicaciones para el binomio madre-producto ^2^. Para el producto se asocia con edad prematura (<37 semanas), bajo peso al nacer y APGAR con puntuación <7 ^5^^-^^7^. De igual forma, para la mujer adolescente se incrementa riesgo de complicaciones obstétricas, entre los que se encuentran preeclampsia-eclampsia, hemorragia posparto, endometritis puerperal, infecciones sistémicas, mayor uso de episiotomía y desgarros perineales durante el parto, estos últimos son la lesión obstétrica más frecuente ^7^^-^^9^. 

Se reconoce como factores de riesgo para desgarros perineales la primíparidad ^10^, pues hasta el 73% presentan un desgarro perineal moderado y entre el 1%-19% de los partos vaginales cursan con laceración esfinteriana, involucrando desgarros de tercer y cuarto grado. Otro factor es un IMC elevado, pues esto incrementa el riesgo de macrosomía y de parto instrumentado ^11^, en el cual se corre mayor riesgo si se utiliza fórceps en comparación con la utilización de ventosas ^12^. En cuanto a la episiotomía, su realización durante el parto incrementa la cantidad de desgarros perineales severos, siendo menos dañina la técnica mediolateral ^13^. Específicamente en parto adolescente se han documentado factores de riesgo para desgarros perineales: primiparidad, posición fetal, diabetes gestacional que requiere de insulina para el control de la glucosa y duración de la segunda etapa de labor de parto ^14^.

Se estima que en la población general, los desgarros de tercer y cuarto grado ocurren en el 0.8% de todos los partos vaginales en México ^15^. Así mismo se asocian las lesiones obstétricas del esfínter anal, con secuelas a corto y largo plazo, principalmente incontinencia anal, fístulas rectovaginales, dehiscencia de heridas y formación de abscesos, que afectan a las pacientes física y psicológicamente ^11^^,^^16^^-^^18^. Además, las fístulas y la incontinencia fecal ^19^^,^^20^, son en su mayoría, consecuencia de una inadecuada reconstrucción del esfínter, lo que se asocia a problemas médico-legales y elevación de costos en atención en salud ^21^. 

El objetivo de este trabajo es determinar la prevalencia de desgarros de tercer y cuarto grado, en partos adolescentes y población general de un hospital de tercer nivel público en Monterrey, Nuevo León, México. Así mismo en comparación con partos en mujeres adultas, determinar si esta condición supone riesgo para desarrollo de desgarros perineales de alto grado. En comparación con partos en mujeres adultas que desarrollaron desgarros perineales de alto grado, valorar si la adolescencia está relacionada con factores de riesgo para desgarros perineales*.*

## Materiales y Métodos

Se trata de un estudio observacional y analítico. 

### Muestra

Se revisaron expedientes de mujeres con atención obstétrica vaginal en el Hospital Materno Infantil de Alta Especialidad (Monterrey, Nuevo León, México) durante el periodo del 1 de enero hasta 31 de diciembre del 2014. El cual es un centro de referencia que brinda atención obstétrica general, y en especial, de alta complejidad al Noreste de México. Se incluyeron todos los casos, independiente de edad y paridad de la paciente. Debido al enfoque del estudio, sólo se consideraron los casos con desgarros de tercer y cuarto grado, ya que en estos grados sí existe afectación del esfínter anal. Sin embargo, se excluyeron los casos con presentaciones diferentes a la cefálica, gestaciones múltiples, nacimiento por cesárea o antes de las 36 semanas de gestación (parto prematuro). De estos casos se documentó lo siguiente: instrumentación del parto, realización de episiotomía, peso del producto, índice de masa corporal de la madre al momento del parto y paridad de la paciente. 

### Análisis estadístico 

Se dividió al total de pacientes en mujeres adultas aquellas con edad mayor o iguales de 19 años y mujeres adolescentes aquellas menores 19 años. Para todas las comparaciones se usó a las mujeres adultas. El análisis se realizó mediante el software SygmaPlot (Systat software versión 12). La prevalencia se calculó en relación con el total de partos por vía vaginal para esos rangos de edad en el año 2014, estos datos fueron obtenidos mediante el censo del hospital. Para la presentación de los resultados de las variables cualitativas se utiliza la cantidad de pacientes con la característica positiva y el porcentaje relativo. Las variables continuas se presentan con mediana y rango de los datos. Para determinar la asociación con riesgo se calcula la razón de momios sin ajustar. Se usó la prueba exacta de Fisher para comparar variables cualitativas y la prueba de U de Mann-Whitney para los contrastes entre variables cuantitativas. Se consideró como significancia estadística cuando el estadístico *p* ≤0.05. 

### Consideraciones éticas 

Este trabajo cuenta con aprobación de los comités de ética del Hospital Materno Infantil de Alta Especialidad (Monterrey, Nuevo León, México) y Escuela de Medicina del Tecnológico de Monterrey. Todos los datos fueron recopilados de forma anónima. La información recabada para el estudio fue con fines exclusivos de investigación y únicamente los investigadores involucrados tuvieron acceso. 

## Resultados

De los 13,882 nacimientos reportados en el hospital durante el año 2014, se realizaron 8,847 partos vaginales, de los cuales 2,404 (27.2%) fueron en mujeres adolescentes. Se obtuvo un total de 179 casos de pacientes con desgarros de tercer y cuarto grado para el análisis, esto representó una prevalencia general del 2.0% de desgarros de tercer y cuarto grado en un año en mujeres atendidas por parto vaginal. De los grupos en los que se dividió la muestra, el grupo de mujeres adolescentes incluyó 60 casos (33.5%), esto es una prevalencia de 2.5% de desgarros en todos los partos vaginales en adolescentes atendidos durante un año en la institución. Estos resultados pueden verse resumidos en la Figura 1. El riesgo de desgarros de alto grado fue mayor en partos adolescentes que en adultas (razón de momios= 1.36, IC 95%= 0.99-1.86, *p*= 0.05). 

**Figura 1:** Flujograma de selección de casos

En la Tabla 1 se presentan los resultados de la comparación de los factores de riesgo en los partos con desgarro de alto grado en adolescentes contra adultas. No se encontró diferencia significativa al comparar las variables recopiladas. 

**Tabla 1:** Comparación de pacientes con desgarros de alto grado para variables de interés

Variables	Grupos de pacientes		
	Adolescentes	Adultos	Población general con desgarros de alto grado
	(<19 años)	(≥19 años)	
Casos	60 (33.5%)	119 (66.5%)	179 (100%)
Edad	17 (14-18)	22 (19-40)	20 (14-40)
Primiparidad	56 (93.3%)	83 (69.7%)	139 (77.7%)
Parto instrumentado	48 (80.0%)	88 (74.0%)	136 (76.0%)
Realización episiotomía	56 (93.3%)	103 (86.6%)	159 (88.8%)
IMC	25.13 (17.80-39.43)	25.89 (18.90-48.39)	25.61 (17.80-48.39)
Peso producto (g)	3,390 (2,230-4,060)	3,270 (2,250-4,390)	3,300 (2,230-4,390)
Desgarros 3er grado	54 (90.0%)	108 (90.8%)	162(90.5%)
Desgarros 4to grado	6 (10.0%)	11 (9.2%)	17 (9.5%)

## Discusión

En este trabajo, se encontró una prevalencia mayor a lo reportado de desgarros perineales de alto grado durante el parto en la población general en comparación con la prevalencia de 0.8% en partos vaginales en población general mexicana en otro estudio ^15^. Este último trabajo recopila la experiencia de 21 centros en México, representando una mayor muestra de la población mexicana. Sin embargo, en cinco de estos centros, no se reportaron laceraciones obstétricas de tercer y cuarto grado, lo que pudiera suponer un subreporte. Como una posible explicación para la mayor prevalencia en el presente trabajo, es importante resaltar que la información se basa en un solo centro. Además, pudiendo estar relacionado con sesgo de selección pues es una unidad de referencia para atención obstétrica de alta complejidad para la población con servicios de salud públicos en el noreste del país. Pesé a estos posibles sesgos, es importante reportar que al menos, en la población atendida, la prevalencia de desgarros perineales es mayor. Se proponen estudios que comparen la experiencia y permitan caracterizar mejor las diferencias entre las prevalencias y factores de riego de laceraciones perineales obstétricas entre varias regiones de nuestro país. 

Existe poca evidencia que estudie a la adolescencia como factor de riesgo para desarrollo de desgarros de alto grado. En la búsqueda bibliográfica llevada a cabo para la elaboración de este documento no se encontraron estudios que determinaran en población mexicana la adolescencia como factor de riesgo para este tipo de patología. La experiencia en otras poblaciones apoya a la adolescencia como factor de riesgo para este tipo de complicación ^22^, aunque existe evidencia que sugiere lo contrario ^15^^,^^23^^,^^24^.

Como limitación de este trabajo no es posible realizar un análisis donde se pueda determinar la adolescencia como factor de riesgo independiente para el desarrollo de desgarros de alto grado durante el parto. Además, si bien el resultado es estadísticamente significativo, el intervalo de confianza de la razón de momios cruda es muy amplio y se encuentra por debajo de la unidad. Aún así, los datos permiten sugerir que se trata de un grupo propenso para este tipo de patología y recomendar a los clínicos atención durante la atención obstétrica vaginal de este grupo de edad para evitar esta complicación

## Conclusiones

En esta población hay una mayor prevalencia de desgarros respecto a lo reportado en otros estudios en población mexicana. Las diferencias en las prevalencias encontradas pudieran estar con relación a un pobre reconocimiento de esta complicación o un sesgo de selección. Los datos encontrados instan a dar más importancia a esta complicación. Así mismo, los datos sugieren que la adolescencia pudiera ser un factor de riesgo para desgarros de alto grado. Es importante resaltar la poca literatura sobre desgarros perineales enfocados a la población adolescente y mexicana. Por lo que, presenta limitaciones, contribuye y permite aportar en un área en la que existe poca información. Las implicaciones prácticas de estos hallazgos es de reconocer a los partos adolescentes como un grupo vulnerable para el desarrollo de desgarros perineales de alto grado. De esta forma, justificando el desarrollo de programas durante la atención obstétrica vaginal enfocados a disminuir la prevalencia de desgarros perineales de alto grado en nuestra población y con especial énfasis a las adolescentes.
